# Type I Interferon Pathway Activation Disrupts Monocyte Maturation and Enhances Immune Evasion in Multiple Myeloma

**DOI:** 10.1002/advs.202510816

**Published:** 2025-11-30

**Authors:** Jian Cui, Jingwei Wang, Xiaoyun Li, Lina Wang, Xuehan Mao, Rui Lyv, Wenqiang Yan, Jingyu Xu, Jieqiong Zhou, Chenxing Du, Shuhui Deng, Mu Hao, Yan Xu, Shuhua Yi, Dehui Zou, Tao Cheng, Xin Gao, Lugui Qiu, Gang An

**Affiliations:** ^1^ State Key Laboratory of Experimental Hematology National Clinical Research Center for Blood Diseases Haihe Laboratory of Cell Ecosystem Institute of Hematology & Blood Diseases Hospital Chinese Academy of Medical Science & Peking Union Medical College Tianjin 300020 China; ^2^ Tianjin Institutes of Health Science Tianjin 301600 China; ^3^ LeBow Institute for Myeloma Therapeutics and Jerome Lipper Multiple Myeloma Center Dana‐Farber Cancer Institute Harvard Medical School Boston MA USA; ^4^ Department of Hematology Beijing Tsinghua Changgung Hospital School of Clinical Medicine Tsinghua Medicine Tsinghua University Beijing 102218 China; ^5^ Beijing GoBroad Boren Hospital Beijing China

**Keywords:** monocyte, multiple myeloma, single‐cell RNA sequencing, tumor microenvironment

## Abstract

Monocyte‐derived cells, including osteoclasts, dendritic cells, and macrophages, are key components of the immunosuppressive tumor microenvironment in multiple myeloma (MM). However, the mechanisms linking monocyte dysfunction to immune evasion remain incompletely understood. In this study, single‐cell RNA sequencing (scRNA‐seq) of peripheral blood (PB) and bone marrow (BM) monocytes was performed from healthy donors (HDs) and MM patients to generate a comprehensive single‐cell transcriptional map. Although PB and BM monocytes displayed comparable cellular compositions, MM monocytes exhibited marked transcriptional alterations, most prominently within the type I interferon (IFN) signaling pathway. Trajectory analyses revealed IFN‐driven disruptions in monocyte differentiation and developmental trajectories in both PB and BM compartments. Functional co‐culture assays demonstrated that activation of the type I IFN pathway enhanced MM cell proliferation, suggesting that IFN‐mediated monocyte reprogramming facilitates tumor progression. In an independent validation cohort, longitudinal sampling before and after induction therapy confirmed that anti‐myeloma treatment alleviated the excessive IFN response of BM monocytes. Collectively, these findings uncover a mechanistic link between aberrant IFN activation and monocyte dysregulation in MM, providing new insights into immune dysfunction and highlighting the IFN pathway as a potential therapeutic target to restore anti‐tumor immunity.

## Graphical abstract

This study shows that monocytes in multiple myeloma display an excessive interferon response, leading to transcriptional reprogramming and altered differentiation. Using single‐cell sequencing, coculture experiments, and patient samples before and after therapy, the authors demonstrate that induction treatment reduces this interferon response, highlighting monocyte dysfunction as a driver of the immunosuppressive tumor microenvironment in multiple myeloma.

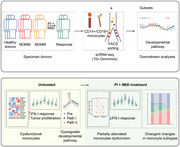

## 1 Introduction

The progression of multiple myeloma (MM) involves a series of steps that encompass the accumulation of cytogenetic abnormalities and transcriptional changes within malignant plasma cells (PCs). Nevertheless, the aberrant interactions between PCs and the tumor microenvironment (TME) also hold considerable importance in the pathogenesis of MM.^[^ ^1^, ^2^ ^]^ Extensive evidence suggests that both hematopoietic cells and non‐hematopoietic cells, including regulatory T cells (Tregs),^[^ ^3^ ^]^ regulatory B cells (Bregs),^[^ ^4^ ^]^ myeloid‐derived suppressor cells (MDSCs),^[^ ^5^, ^6^ ^]^ and stromal cells,^[^ ^7^ ^]^ contribute to PC proliferation. Recent advancements in single‐cell RNA sequencing (scRNA‐seq) technology have provided valuable insights into the compositional and transcriptional alterations within the MM TME.^[^ ^7^, ^8^, ^9^, ^10^, ^11^, ^12^, ^13^, ^14^, ^15^ ^]^ A previous study has indicated that compromise of the immune microenvironment occurs even in precursor stages of MM. This compromise is characterized by a specific accumulation of Tregs and a decline in CD8^+^ memory populations within the MM TME.^[^ ^8^ ^]^ Additionally, a more recent study has observed changes in immune cell populations as MM progresses, including an increase in CD8^+^ effector T cells and Tregs.^[^ ^11^ ^]^ However, previous scRNA‐seq studies have predominantly focused on adaptive immune cells, while the quantitative and qualitative alterations of innate immune cells, particularly monocytes, within the MM TME have received less attention.

Human monocytes, as crucial components of the innate immune response, can be categorized into three main subsets based on the surface expression of CD14 and CD16: classical (CD14^++^CD16^−^), intermediate (CD14^++^CD16^+^), and non‐classical (CD14^+^CD16^++^) monocytes.^[^ ^16^, ^17^ ^]^ Emerging evidence from scRNA‐seq studies has revealed the dysregulation of monocytes in inflammatory disorders such as acute respiratory syndrome,^[^ ^18^ ^]^ and COVID‐19.^[^ ^19^, ^20^, ^21^ ^]^ In hematological malignancies like B cell acute lymphoblastic leukemia, scRNA‐seq has demonstrated an abundance of non‐classical monocytes at disease diagnosis.^[^ ^22^ ^]^ Furthermore, various monocyte‐derived cells, including osteoclasts,^[^ ^23^ ^]^ dendritic cells (DCs),^[^ ^24^ ^]^ and macrophages,^[^ ^25^, ^26^ ^]^ have been implicated in immunosuppression within the MM TME. These cells can be functionally reprogrammed by local cues within the MM TME and contribute to chemotherapy resistance and disease progression.^[^ ^27^, ^28^, ^29^ ^]^ However, the relationship between monocytes themselves and immunosuppression in the context of MM, despite being the primary source of these long‐lived immunosuppressive cells, remains less explored.^[^ ^30^ ^]^ Additionally, limited information is available regarding the functional and developmental alterations of monocytes within the MM TME based on comprehensive scRNA‐seq studies.

To address these knowledge gaps, we performed a comprehensive analysis by generating a single‐cell map of peripheral blood (PB) and bone marrow (BM) monocytes from both healthy donors (HDs) and MM patients. Our findings revealed that MM monocytes exhibited an augmented type‐I interferon (IFN) response compared to monocytes from HDs, while showing relatively limited changes in overall heterogeneity. Importantly, the excessive type‐I IFN response in MM had a significant impact on the differentiation of both BM and PB monocytes. Further in vitro experiments confirmed that activation of the type‐I IFN signaling pathway could facilitate the proliferation of MM cell line. To investigate the dynamic changes of monocytes over time, we obtained BM monocytes from 10 MM patients at diagnosis and after induction therapy. Through sequencing and analysis, we observed that anti‐tumor therapy successfully mitigated the excessive type‐I IFN response in BM monocytes of MM. Collectively, these results offer novel insights into the transcriptional reprogramming and differentiation landscape of monocytes in MM and uncover a previously underappreciated role of type‐I IFN–driven monocyte dysfunction in shaping the immunosuppressive tumor microenvironment.

## 2 Results

### 2.1 scRNA‐seq Transcriptome Profiling of Monocytes from HDs and NDMM Patients

**Figure 1:**
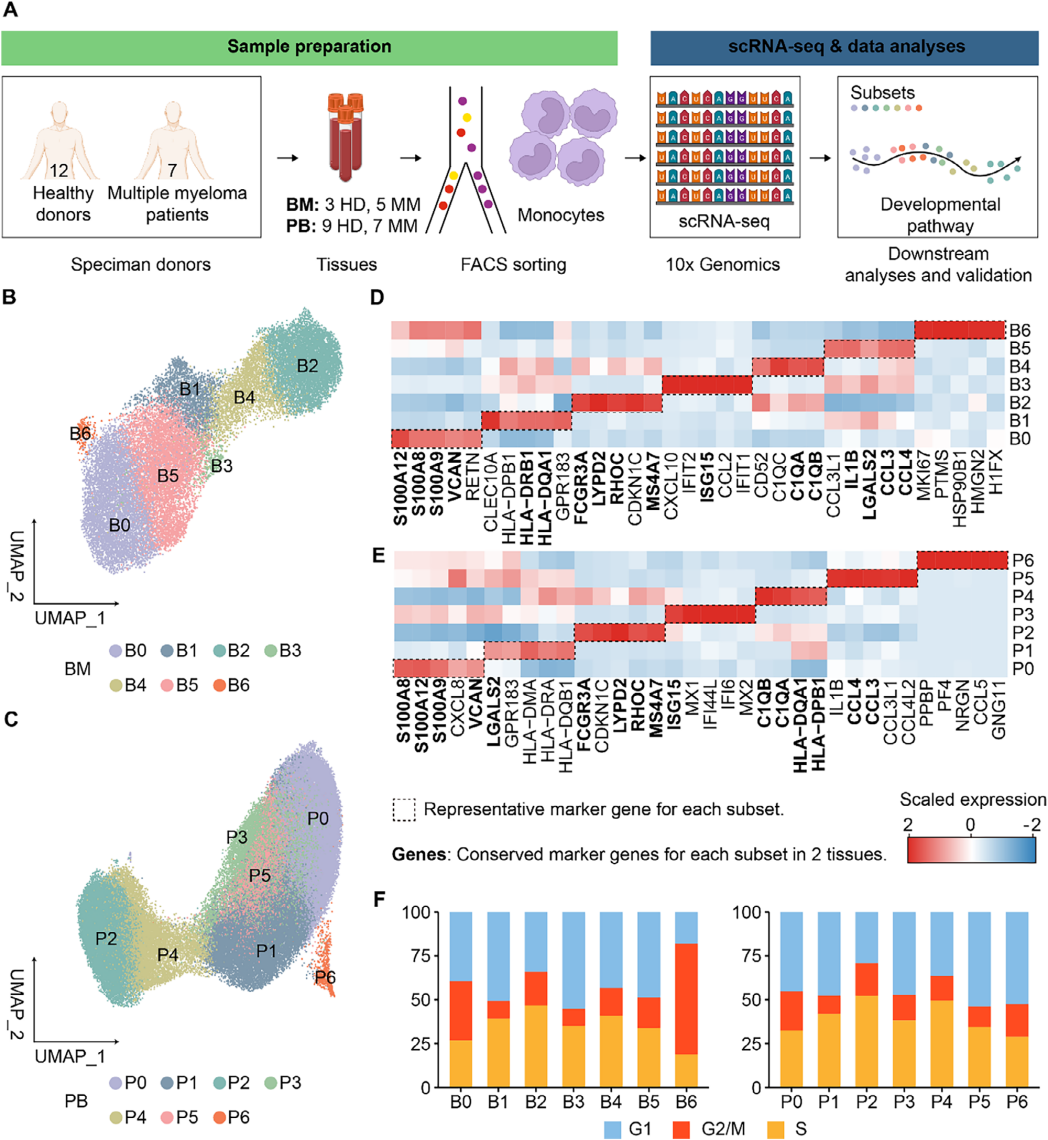
scRNA‐seq transcriptome profiling of monocytes from HDs and NDMM patients. A) A scheme depicting the workflow of this study. BM samples were collected from 3 HDs and 5 NDMM patients, and PB samples were collected from 9 HDs and 7 NDMM patients. Moreover, 10 more patients were included as an internal validation cohort, and BM samples were collected at diagnosis and at remission. B,C) UMAP representations of the integrated single‐cell transcriptomes of 26,683 BM (B) or 82,328 PB (C) monocytes, colored by cell clusters. Each cluster was numbered and labeled with the first letter (s) of the tissue name (B0 for cluster 0 in bone marrow, etc.). D,E) Heatmaps showing the top 10 (by average log twofold change [avgLog2FC]) marker genes for each BM (D) or PB (E) monocyte cluster, excluding the ribosomal and mitochondrial genes. The scaled expression means that the gene expression was centered and scaled among clusters. F) The proportions of BM (upper) or PB (bottom) monocytes in distinct phases of the cell cycle for each cluster.

PB samples were collected from 9 HDs and 7 newly diagnosed MM (NDMM) patients, while BM samples were obtained from 3 HDs and 5 NDMM patients (**Figure** **1**A; Table S1, Supporting Information). Monocytes were sorted from these samples using fluorescence‐activated cell sorting (FACS) based on the expression of cell surface markers CD14 and CD16 (classical monocyte: CD14^++^CD16^–^, intermediate monocyte: CD14^++^CD16^+^, and non‐classical monocyte: CD14^+^CD16^++^) (Figure S1A, Supporting Information).^[^ ^17^, ^31^ ^]^ We performed scRNA‐seq using the 10× Genomics Chromium platform to analyze the purified monocytes from BM and PB samples of HDs and NDMM patients. After stringent quality control measures, a total of 40,160 BM and 90,575 PB single cells were retained and integrated into separate single‐cell datasets (Table S2, Supporting Information). Following data preprocessing and normalization, we identified seven major cell clusters in BM and four major cell clusters in PB using graph‐based clustering (Figure S1B‐E, Supporting Information). To focus specifically on monocyte heterogeneity, we extracted monocytes from the BM and PB single‐cell datasets, resulting in the profiling of 26,683 BM (MM: 7,135 cells; HD: 19,548 cells) monocytes and 82,328 PB (MM: 33892 cells; HD: 48436 cells) monocytes from HDs and NDMM patients.

To further investigate the heterogeneity of monocytes in PB or BM, we conducted unsupervised clustering and categorized PB and BM monocytes into seven clusters, as shown in Figure 1B,C. By utilizing previously established markers for classical, intermediate, and non‐classical monocytes, such as *CD14*, *FCGR3A* (which encodes CD16), and *HLA‐DRA*,^[^ ^31^, ^32^, ^33^ ^]^ along with the top 10 signature genes expressed by each cluster (Figure 1D,E; Figure S2A,B, Supporting Information; Table S3, Supporting Information), we identified CD14^++^ classical monocytes as clusters 0, 1, 3, 5, and 6 (designated as B0, B1, B3, B5, B6 in BM, and P0, P1, P3, P5, P6 in PB). Additionally, CD14^++^CD16^+^ intermediate monocytes were categorized as cluster 4 (B4 in BM and P4 in PB), while CD16^++^ non‐classical monocytes were represented by cluster 2 (B2 in BM and P2 in PB).

Among the classical monocyte subsets, cluster 0 in BM (B0) and PB (P0) exhibited elevated expression of S100A family genes. Cluster 1 in BM (B1) and PB (P1) demonstrated heightened expression of major histocompatibility complex (MHC)‐II‐related genes, including *HLA‐DRA* and *HLA‐DPB1*. Cluster 3 in BM (B3) and PB (P3) displayed high expression of IFN‐stimulated genes (ISG). Cluster 5 in BM (B5) and PB (P5) presented a distinct combination of genes, including chemokine ligands and interleukins (e.g., *CCL3*, *CCL4*, and *IL1B*). B6 in BM represented a cluster characterized by high expression of the *MKI67* gene, while P6 in PB exhibited elevated expression of genes related to megakaryocyte progenitors, such as *PPBP* and *PF4*. A gene ontology (GO) analysis of differentially expressed genes (DEGs) in each monocyte cluster in BM and PB revealed that cluster 1 (B1 and P1) exhibited the highest levels of MHC‐II‐related genes and pathway expression, indicating their robust antigen processing and presentation capacities among monocytes (Figure S2C,D, Supporting Information). Additionally, the cell cycle profiles were similar across all monocyte clusters in PB and BM, except for B6, which showed the highest proportion of cells in the S + G2/M phase (Figure 1F).

### 2.2 Tissue‐Specific Transcriptome Features

The expression of classical and non‐classical monocyte markers, as defined by previous scRNA‐seq studies,^[^ ^31^, ^33^ ^]^ were also highly enriched in the monocyte clusters of HDs (Figure S3A,B, Supporting Information). Further analysis revealed a significantly higher proportion of cluster 5 in BM compared to PB in HDs (26.6% vs. 10.4%), while a higher proportion of monocytes in cluster 1 was observed in PB compared to BM in HDs (21.1% vs. 8.0%) (Figure S3C, Supporting Information). Correlation analysis suggested relatively higher intra‐correlations among classical monocytes (clusters 1, 3, and 5) within the same tissue (BM or PB) but relatively lower correlations between monocytes from different tissues (Figure S3D, Supporting Information). Comparative analysis between BM and PB revealed distinct transcriptomic patterns across tissues, indicating that the tissue‐specific transcriptomic features refer to differential gene expression and correlation profiles distinguishing monocyte subsets between BM and PB.

### 2.3 MM Primes Monocytes for Augmented IFN Response without Affecting their Overall Heterogeneity

**Figure 2:**
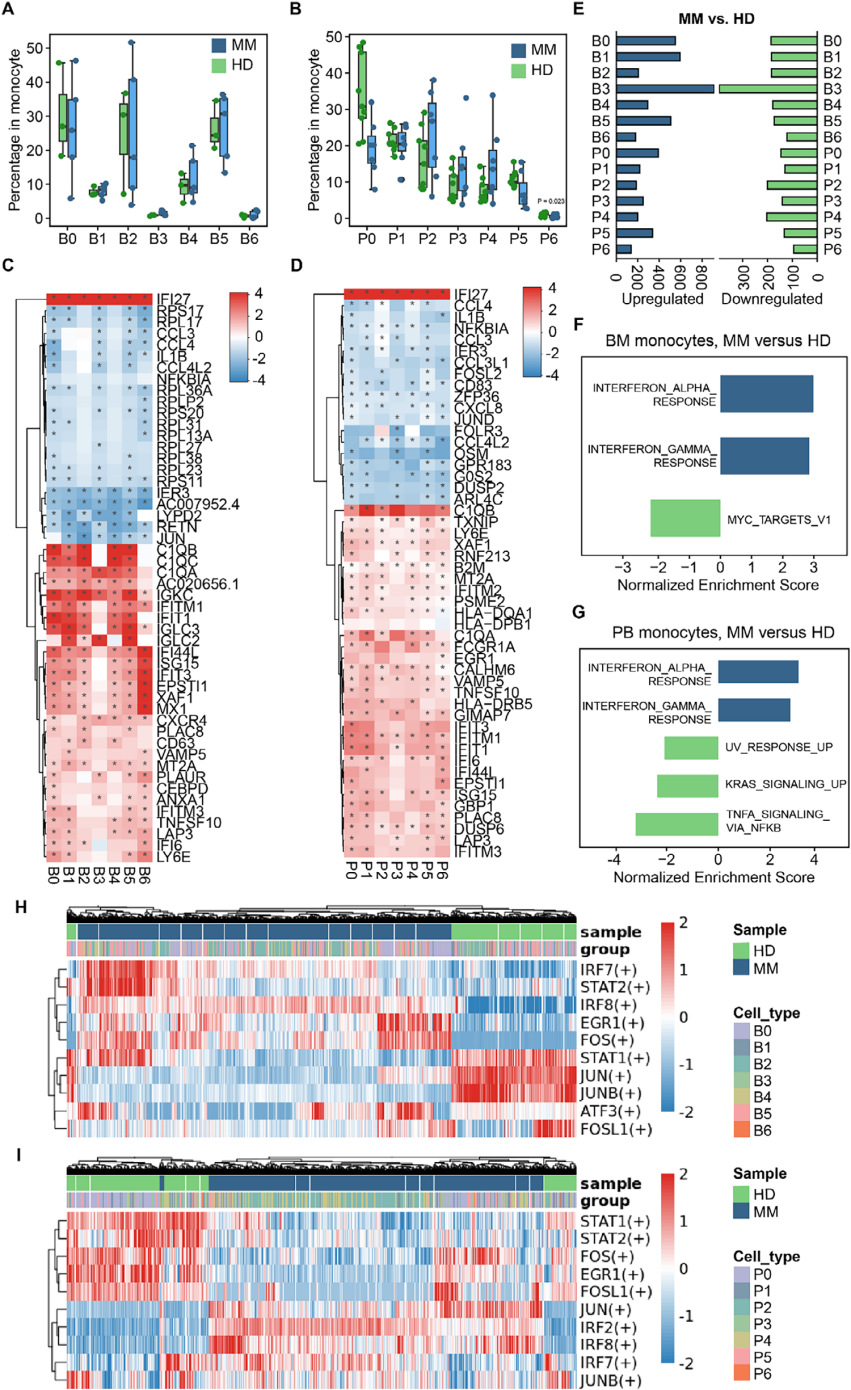
MM primes monocytes for augmented interferon response without affecting their overall heterogeneity. A,B) Boxplots comparing proportions of 7 BM (A) or PB (B) monocyte clusters between HD and MM. Wilcoxon rank‐sum test was used to measure the differences between the two groups. Horizontal lines in the boxplots represent the median, the lower and upper hinges correspond to the first and third quartiles, and the whiskers extend from the hinge up to 1.5 times the interquartile range from the hinge. Significance was calculated using the Wilcoxon rank‐sum test (two‐sided). C,D) Heatmaps showing the Log2FC in gene expression of the top 50 DEGs in BM (C) and PB (D) monocytes between MM patients and HDs. The asterisks mean absolute Log2FC > 1 in corresponding monocyte clusters. E) Bar plot showing the number of upregulated (left) or downregulated (right) genes between MM and HD for each monocyte cluster. F,G) Hallmark pathway enrichment analyses of the DEGs between MM and HD in BM (F) or PB (G) monocytes. Selected GO terms with Benjamini–Hochberg‐corrected *p*‐values < 0.05 (one‐sided Fisher's exact test) are shown. H,I) Heatmaps of the scaled AUC scores of expression regulation by TFs, as estimated using SCENIC, for each of the BM (H) and PB (I) monocytes. Shown are 10 selected TFs.

To identify MM‐specific alterations in monocytes, we compared the proportions of each BM or PB monocyte cluster between HDs and MM patients. Considerable heterogeneity in monocyte composition was observed in both groups (Figure S4A, Supporting Information). Nevertheless, apart from a lower proportion of the P6 cluster in MM, the overall monocyte composition appeared largely comparable between MM patients and HDs (**Figure** **2**A,B).

To further explore the transcriptional alterations of monocytes specific to MM, we performed DEGs analysis between HDs and MM patients. Pseudo‐bulk differential expression analysis revealed several genes expressed at higher levels in MM compared to HDs, including IFN pathway‐related factors *ISG15* and *IFIT1* in both BM and PB monocytes, consistent with previous scRNA‐seq data.^[^ ^8^, ^34^ ^]^ Conversely, the expression of cytokines and cytokine receptors was generally higher in HD compared to MM for both BM and PB monocytes, with significant differences observed for *CCL3*, *CCL4*, and *IL1B*, which play roles in inflammatory signaling. Additionally, several small and large ribosome unit genes, such as *RPL17*, *RPL23*, and *RPS11*, were downregulated in BM MM monocytes (Figure 2C,D; Table S4, Supporting Information). Similar results were obtained when accounting for the monocyte cluster (Figure S4B‐G, Supporting Information). These findings suggest that monocytes in MM display a reduced pro‐inflammatory cytokine program and impaired ribosomal gene expression, which may reflect compromised immune activation and altered protein synthesis capacity in the disease context.

Furthermore, DEGs analysis revealed a higher number of upregulated DEGs observed in BM monocytes compared to PB monocytes in the MM versus HD comparison, while the B3 cluster exhibited the largest number of DEGs among all monocyte clusters (Figure 2E), indicating MM microenvironment‐related phenotype in monocytes from MM patients.

Rank‐based gene set enrichment analysis (GSEA), comparing MM monocytes to HD monocytes across tissues, demonstrated significant enrichment for the IFN‐related pathway, specifically the ‘Interferon alpha response’ and ‘Interferon gamma response’ (Figure 2F,G). Moreover, the ISG‐related signature tended to be enriched in MM compared to HD for all monocyte clusters, further supporting an overall heightened IFN response in monocytes of MM patients versus HDs (Figure S4H,I, Supporting Information).

Finally, we explored transcription factors (TFs) in monocytes that may regulate IFN‐associated transcriptional programs. Single‐Cell Regulatory Network Inference and Clustering (SCENIC) analysis revealed that genes regulated by IRF7 and IRF8 TFs were upregulated in almost all MM monocytes, while genes downregulated were regulated by STAT1 (Figure 2H,I). Notably, IRF7 plays an important role in regulating IFN‐dependent immune responses, while STAT1 can promote inflammatory responses in monocytes and their differentiation into macrophages.^[^ ^35^, ^36^ ^]^ These findings supported an augmented IFN response in monocytes of MM patients and suggested potential TFs that might transcriptionally regulate MM‐related activation of the IFN signaling transduction pathway.

### 2.4 Excessive IFN Response in MM Alters the Differentiation Paths of BM Monocytes

**Figure 3:**
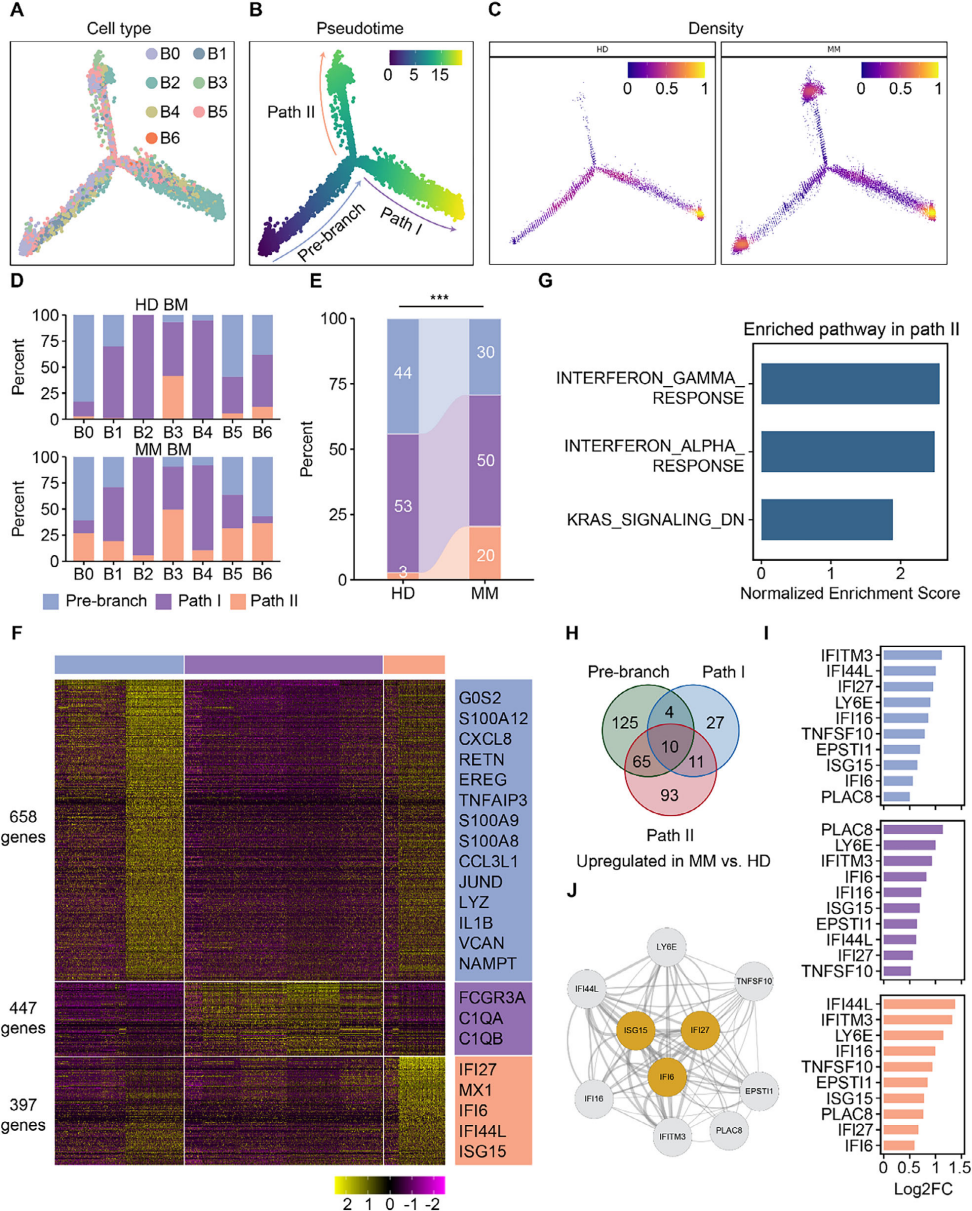
Excessive IFN response in MM alters the differentiation paths of BM monocytes. A‐C) Trajectories predicted using the Monocle2 software for BM monocytes from HDs and MM patients. Cells are color‐coded by monocyte clusters (A), by the pseudotime trajectory (B), and by cell density (C). D) Bar plots show the distribution of BM monocyte clusters in different differentiation paths from HD (upper) and MM (bottom), respectively. E) Bar plot comparing proportions of monocytes in three BM differentiation paths, color‐coded by differentiation paths, and corresponds to Figure 4D. ***p‐value < 0.001, by two‐sided χ^2^ test. F) Heatmap showing scaled expression of discriminative gene sets for BM monocytes for each differentiation path with average log 2‐fold change ≥ 0.25 (avgLog2FC ≥ 0.25). Color bars in the right margin highlight gene sets of interest. G) Hallmark pathway enrichment analysis of the upregulated genes in BM Path II cells compared with monocytes in other differentiation paths. H) The number of shared upregulated genes in MM versus HD for each BM differentiation path. I) Bar plots of the 10 shared upregulated DEGs in MM versus HD for all three BM differentiation paths. DEGs are ranked by the avgLog2FC along the y‐axis. Color‐coded by differentiation paths. J) STRING network analysis of the 10 shared upregulated DEGs in MM versus HD in relation to *ISG15*, *IFI6*, and *IFI27*. Line thickness indicates confidence.

To explore the transitional and differentiation relationships among BM monocytes, we integrated monocytes from both MM and HD, and conducted pseudotime analysis and reconstructed differentiation paths using Monocle2 (**Figure** **3**A–C).^[^ ^37^ ^]^ Under steady‐state conditions, the pseudotime differentiation axis started from B0 and other classical monocyte clusters, leading to Path I comprising B4 and B2 (Figure 3D; Figure S5A, Supporting Information). This differentiation path from the Pre‐branch to Path I corresponded to the process of classical monocytes differentiating into intermediate monocytes and non‐classical monocytes. There were numerous overlapping marker genes between the Pre‐branch and B0 cluster, as well as between Path I and the B2 cluster (Figure S5B, Supporting Information).

Remarkably, we observed a distinct differentiation path from the Pre‐branch into Path II that was predominantly present in MM BM (Figure 3D,E). We then identified signature genes and enriched pathways for each differentiation path (Table S5, Supporting Information). Notably, the signature genes of Path II included several IFN pathway‐related factors, such as *IFIT1*, *IFI27*, *MX1*, *IFI6*, *IFI44L*, and *ISG15* (Figure 3F; Table S5, Supporting Information). Ranked GSEA for Path II demonstrated significant enrichment for the ‘Interferon gamma response’ and ‘Interferon alpha response’ pathway (Figure 3G). Furthermore, correlation analysis indicated the conservation of Path I between HD and MM but not of the Pre‐branch and Path II (Figure S5C, Supporting Information). We hypothesized that specific molecular pathways contributed to the altered differentiation of MM monocytes and conducted differential expression analysis for each differentiation path between MM and HD. We identified 10 shared upregulated DEGs in MM compared to HD, primarily consisting of IFN‐related factors, including *IFI27*, *ISG15*, *IFI6*, and *IFI44L* (Figure 3H,I; Table S6, Supporting Information). Notably, significant enrichment for the ‘Interferon alpha response’ and ‘Interferon gamma response’ pathways was observed in MM Path II compared to HD Path II (Figure S5D, Supporting Information). Furthermore, additional analysis using available coexpression databases through STRING demonstrated that *ISG15*, *IFI6*, and *IFI27* signaling were associated with all of the shared upregulated DEGs in MM versus HD (Figure 3J).^[^ ^38^ ^]^ To investigate the impact of excessive IFN response in MM on the differentiation of BM monocytes, we compared the functional differences between MM and HD for each differentiation path. Interestingly, we observed a positive correlation between BM monocyte differentiation and IFN response (Figure S5E, Supporting Information). Moreover, in all three differentiation paths, there was an upregulated expression of IFN response gene set, with the most pronounced alterations observed in clusters (Figure S5F,G, Supporting Information). Collectively, our findings suggest that the excessive IFN response in MM may contribute to altered differentiation of bone marrow monocytes.

### 2.5 Alterations of PB Monocyte Differentiation Paths in MM

Consistent with the differentiation path observed in HD BM monocytes, the P0 cluster was identified as the starting point of the HD PB differentiation trajectory, while the P4 (intermediate monocytes) and P2 (non‐classical monocytes) clusters were found only in the later stage (Figure S6A–D, Supporting Information). A similar IFN‐driven Path II was also observed in MM PB monocytes (Figure S6D–G, Supporting Information). Additionally, we identified three shared upregulated DEGs in MM compared to HD for each PB differentiation path, namely *IFITM3*, *PLAC8*, and *VAMP5* (Figure S6H, Supporting Information; Table S7, Supporting Information). Among these genes, *IFITM3* is known to be an interferon‐inducible antiviral factor, *PLAC8* has been implicated in immune regulation and differentiation processes, and *VAMP5* encodes a vesicle‐associated membrane protein involved in vesicular trafficking and immune responses. In summary, our results indicated that excessive IFN response similarly resulted in alterations of PB monocyte differentiation paths in MM.

### 2.6 in vitro Analysis of IFN Response Activation in MM TME

**Figure 4:**
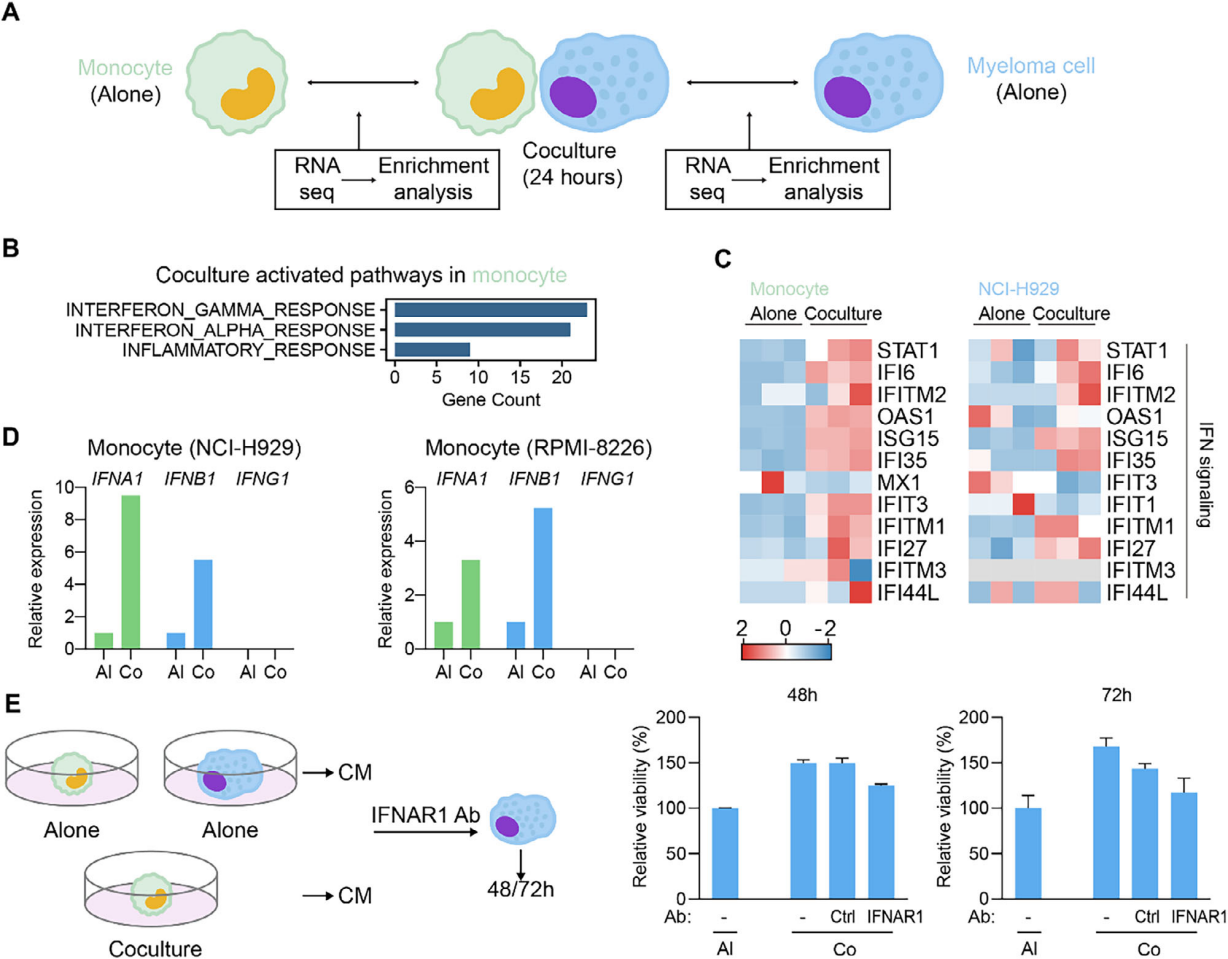
In vitro analysis of IFN response activation in MM TME. A) Schematic diagram of the in vitro coculture experimental setup using human monocyte cell line (THP‐1) and MM cell lines. THP‐1 cell line and MM cell lines were cocultured for 24 h before cell separation for RNA‐sequencing. B) RNA‐sequencing analysis comparing MM‐induced changes in monocytes (RNA‐sequencing analysis comparing MM‐induced changes in monocytes (n = 3 biologically independent samples). C) Heatmaps of genes in IFN signaling pathway in monocytes and MM cells (n = 3 biologically independent samples). D) Expression of IFN genes in cocultured cells, showing induction of type I IFNs (*IFNA1* and *IFNB1*) but not type II IFN (*IFNG1*) in monocytes (n = 3 biologically independent samples). E) Conditioned media (CM) collected from cocultures promoted MM cell proliferation compared to monoculture CM, and this effect was abolished by neutralizing antibodies against the type I IFN receptor, IFNAR1, with IgG isotype antibody used as control (n = 3 biologically independent samples, mean ± s.e.m.).

To better understand the mutual interactions between monocytes and MM cells, we established in vitro coculture experiments and performed RNA sequencing (RNA‐seq) on both cell types (**Figure** **4**A). We utilized the THP‐1 monocyte cell line together with the human MM cell lines NCI‐H929 and RPMI‐8226. Monocytes and MM cells were cocultured at a 1:1 ratio for 24 h before separation and RNA‐sequencing. Compared to cells cultured alone, we conducted DEGs analysis of MM cell–induced transcriptional changes in monocytes, as well as monocyte‐induced changes in MM cells. Notably, the IFN signaling pathway emerged as the top activated pathway in both monocytes and MM cells (Figure 4B,C; Figure S7A,B, Supporting Information). We next assessed the expression of IFN‐related genes in our in vitro coculture system. Human monocytes were found to express type I IFNs (*IFNA1* and *IFNB1*) but not type II IFN (*IFNG1*) (Figure 4D). Finally, we investigated whether IFN pathway activation in monocytes, particularly the production of IFN‐α, contributed to the formation of an immunosuppressive TME. Conditioned media (CM) were collected after 72 h from both monocultures (MM cells or monocytes alone) and cocultures of MM cells with monocytes. Treatment with coculture‐derived CM significantly promoted the proliferation of MM cell lines compared to monoculture‐derived CM. Furthermore, this enhanced proliferation was abolished by the addition of neutralizing antibodies against the type I IFN receptor, IFNAR1 (Figure 4E).

### 2.7 Anti‐Tumor Therapy Overcomes Excessive IFN Response of BM Monocytes of MM

**Figure 5:**
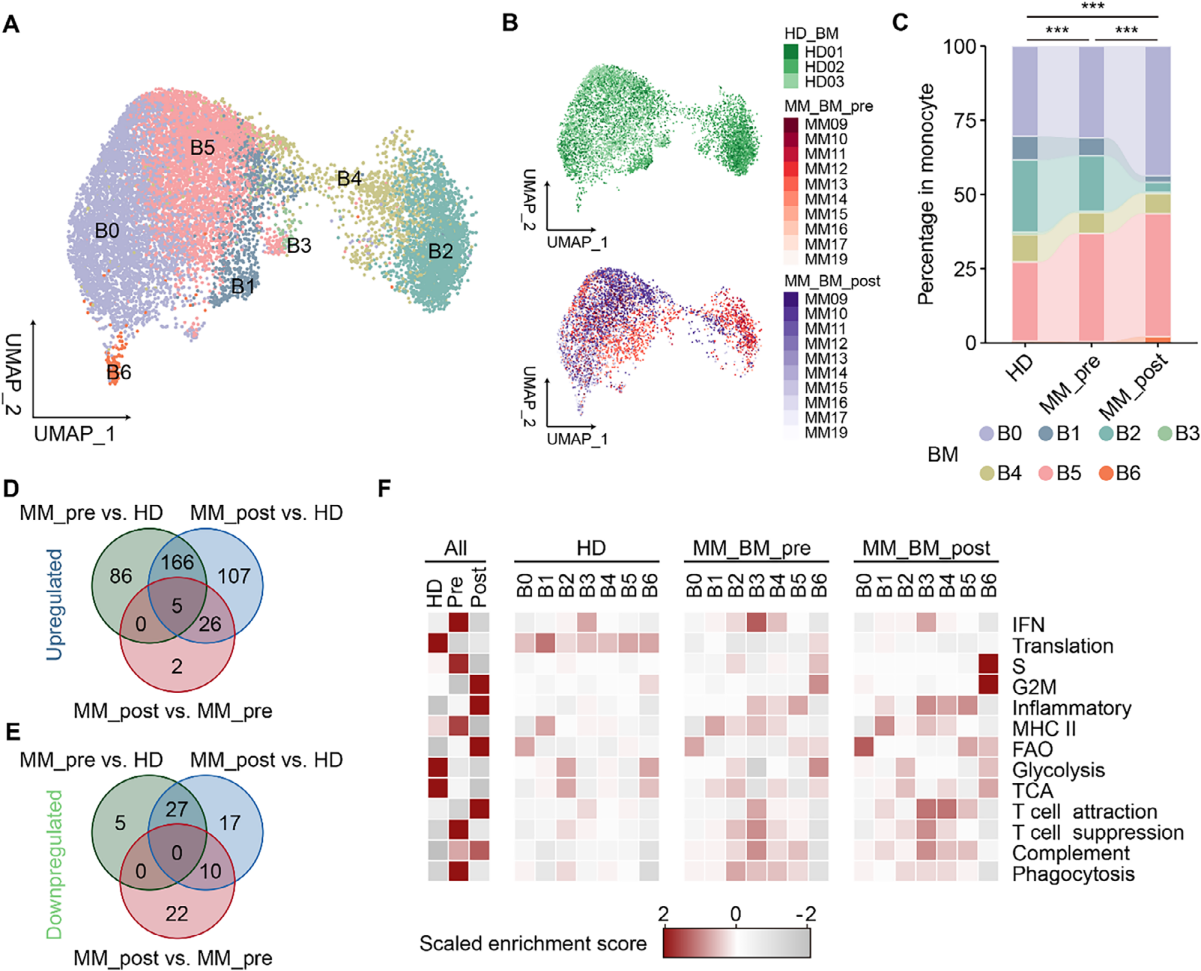
Anti‐tumor therapy overcomes excessive interferon response in BM monocytes. A) UMAP visualization of BM monocytes from HDs and MM patients at diagnosis and after induction therapy, colored by monocyte clusters. B) UMAP visualization of BM monocytes from HD (upper) and paired MM samples at diagnosis (MM_BM_pre) and after induction therapy (MM_BM_post) (bottom), color‐coded by samples. C) Bar plot comparing compositions of BM monocyte clusters from HDs, MM patients at diagnosis (MM_pre) and after induction therapy (MM_post), color‐coded by monocyte clusters. ***p‐value < 0.001, by two‐sided χ^2^ test. D) The number of shared upregulated DEGs in HD, MM_pre, and MM_post compared with each other. E) The number of shared downregulated DEGs in HD, MM_pre, and MM_post compared with each other. F) Heatmap showing the scaled enrichment scores of monocyte feature pathways for each BM monocyte cluster from HD, MM_pre, or MM_post.

To investigate whether anti‐myeloma induction therapy could overcome the excessive IFN response of BM monocytes in MM, we included additional 10 patients at diagnosis and after 2‐4 cycles of induction therapy, and nine of these cases received a proteasome inhibitor plus an immunomodulatory drug induction. The clinical characteristics of these patients are provided in Table S8 (Supporting Information). This cohort serves as an independent internal validation cohort for our previous findings. BM samples were obtained, and after performing scRNA‐seq and data processing, a total of 2489 and 3640 monocytes from MM at diagnosis and after induction therapy passed quality control, respectively, and were identified using monocyte markers, including *CD14*, *FCGR3A* (CD16), *LYZ*, and *CST3*. These monocytes were included in downstream data analysis. Integrated analysis with BM monocytes from HDs revealed the same 7 BM monocyte clusters in MM monocytes (**Figure** **5**A,B).

We first compared the compositions of BM monocytes between different groups. Compared with HDs and MM patients at diagnosis, MM patients after induction therapy displayed a higher proportion of B0 and B5 cluster, while the B2 cluster (non‐classical monocytes) was significantly reduced (Figure 5C). Furthermore, pseudo‐bulk differential expression analysis revealed a greater number of overlapping upregulated and downregulated DEGs between MM at diagnosis versus HD and MM after induction therapy versus HD, compared to MM at diagnosis versus MM after induction therapy (Figure 5D,E; Tables S9 and S10, Supporting Information). These findings indicated that the core transcriptional alterations remained unchanged after induction therapy. We then compared the potential functional differences for each BM monocyte cluster across different samples, specifically MM at diagnosis and MM after induction therapy. Interestingly, after induction therapy, MM patients exhibited upregulated expression of inflammatory, fatty acid oxidation, T cell attraction, and complement gene sets in BM monocyte clusters. On the other hand, MM at diagnosis showed the highest expression of the IFN response gene set (Figure 5F). Differential expression analysis further revealed that BM monocytes of MM after induction therapy displayed higher expression of chemokine ligands and interleukins, including *CCL3*, *CCL4*, *IL1B*, and *CXCL8*, indicating an enhanced inflammatory profile (Figure S8A, Supporting Information). In contrast, MM at diagnosis showed higher expression of ISGs, such as *ISG15*, *IFI6*, and *IFI44L*, reflecting a stronger IFN response (Figure S8B, Supporting Information). Furthermore, the expression of IFN response gene set was downregulated in almost all BM monocyte clusters of MM after induction therapy, suggesting a decrease in IFN response (Figure S8C, Supporting Information). Similar results were consistently observed in each individual patient after induction therapy, further confirming these findings (Figure S8D, Supporting Information). These results suggested that anti‐myeloma induction therapy led to functional changes in BM monocytes, including reduced IFN response and enhanced T‐cell attraction, potentially contributing to a more favorable immune microenvironment in MM.

In conclusion, our findings demonstrated that anti‐tumor induction therapy effectively mitigated the excessive IFN response observed in MM. These results highlighted the potential of induction therapy in modulating the immune microenvironment of MM.

## 3 Discussion

Multi‐faceted investigations into the heterogeneity of monocytes across different tissues have greatly enhanced our understanding of these cells and their potential clinical implications in various cancer types.^[^ ^33^ ^]^ Building upon previous research, our scRNA‐seq study of monocytes from both HDs and MM patients not only confirmed previous findings but also provided novel insights into the alterations occurring in monocytes during MM.

In our study, we successfully resolved the monocytes from both BM and PB of both HDs and MM patients into seven distinct clusters. These clusters encompassed the classical, intermediate, and non‐classical monocyte populations, which were consistent with previous findings.^[^ ^39^, ^40^, ^41^, ^42^ ^]^ Notably, we observed an increased diversity within the classical monocytes, including clusters characterized by *S100A8* expression (B0 and P0), *HLA* expression (B1 and P1), *CCL3* expression (B5 and P5), and IFN‐related gene expression (B3 and P3). Consistent with the enrichment of MKI67 expression, cells in cluster B6 exhibited the highest proportion of S + G2/M phase cells, indicating that this subset likely represents a proliferative monocyte population within the bone marrow. By analyzing the composition of monocytes under steady‐state conditions, we found a higher frequency of cluster 1 and cluster 3 in the PB. This observation aligned with the notion that PB monocytes circulate in the bloodstream and actively patrol the blood vessel lumen.^[^ ^16^, ^17^ ^]^ On the other hand, BM monocytes exhibit a higher proliferative capacity.^[^ ^43^ ^]^ Our analyses revealed that, despite a reduced proportion of the P6 cluster in MM, the overall distribution of monocyte subsets between MM patients and HDs remained largely comparable. These findings suggest that the primary alterations in MM monocytes are likely to occur at the transcriptional or developmental level rather than being driven by major compositional changes. Such transcriptional reprogramming may therefore underline the dysregulation of monocytes in the MM microenvironment.

Despite the similarities in monocyte composition between MM and HD, our differential expression analysis revealed an excessive IFN response in MM monocytes compared to HD monocytes. Traditionally, the IFN response is recognized for its role in human anti‐tumor immune responses,^[^ ^44^ ^]^ and the expression of the IFN receptor facilitates monocyte migration from the BM to the sites of infection or inflammation.^[^ ^16^ ^]^ However, emerging evidence suggests that IFN can also promote immunosuppression in the TME, representing an immune evasion mechanism employed by tumors to dampen anti‐tumor responses.^[^ ^45^ ^]^ It is noteworthy that chronic exposure to IFN has been associated with the upregulation of PD‐L1 in tumor cells.^[^ ^46^ ^]^ Interactions between IFN and the PD‐1/PD‐L1 axis have been reported, and PD‐L1 has been found to protect tumor cells via inhibitory crosstalk involving the IFN signaling pathway.^[^ ^47^, ^48^ ^]^ Recent studies have also indicated that a low‐level activated IFN microenvironment can promote brain metastasis by enhancing the recruitment of monocytic myeloid cells.^[^ ^49^ ^]^ In the context of scRNA‐seq studies focusing on the MM TME, evidence suggests that IFN‐related gene expression, including *MX1* and *ISG15*, increases in plasma cells during disease progression, with IFN response observed in advanced stages of MM.^[^ ^8^ ^]^ The enhanced IFN‐related gene expression in MM monocytes likely reflects interferon pathway activation within the MM niche, leading to functional reprogramming without measurable changes in monocyte subset proportions.

Consistent with previous reports, our in vitro findings suggest that IFN response activation is linked to a shift toward a less pro‐inflammatory monocyte phenotype and the induction of type I IFNs, which may contribute to an immunosuppressive TME and potentially favor MM cell proliferation. We established coculture systems of monocytes and MM cells and performed RNA‐sequencing, which revealed IFN signaling as the top activated pathway in both cell types. Monocytes selectively expressed type I IFNs (*IFNA1* and *IFNB1*) upon coculture but not type II IFN (*IFNG1*). Functionally, CM from cocultures promoted MM cell proliferation compared to monoculture CM, an effect reversed by neutralizing antibodies against IFNAR1. From a clinical perspective, these results indicate that the IFN–IFNAR1 signaling axis may represent a promising therapeutic target in MM. Selective blockade or modulation of this pathway could potentially reverse monocyte‐mediated immune suppression and attenuate tumor‐supportive inflammatory signaling. However, given the dual roles of IFN in both anti‐tumor immunity and immune tolerance, therapeutic interventions will require careful optimization of dosing, timing, and patient selection to maximize benefit while minimizing potential adverse effects.

The present study also investigated monocyte differentiation paths in the context of MM across tissues. Our findings revealed two distinct differentiation paths for both BM and PB monocytes. Path‐I monocytes were characterized by the expression of *FCGR3A* and *MS4A7* and exhibited numerous overlapping marker genes with the B2 cluster (non‐classical monocytes). The differentiation pathway from Pre‐branch to Path I align with the recognized differentiation process from classical monocytes to non‐classical monocytes, with intermediate monocytes representing a transitional state.^[^ ^43^, ^50^ ^]^ In contrast, in BM, IFN‐related cluster 3 was located at the end of Path II, while in PB, P3 was found at the end of Path II. Importantly, the differentiation path from Pre‐branch to Path II was predominantly observed in MM monocytes, indicating an IFN‐centric perturbation of developmental paths in MM. In the cohort that we longitudinally followed, we observed a decrease in the IFN response across nearly all monocyte clusters in MM BM monocytes after induction therapy. These findings suggested that the reduction in tumor burden following induction therapy may contribute to the alleviation of the chronic or excessive IFN response in MM monocytes within the TME.

Despite the significant findings obtained in our study, there are several potential limitations that should be addressed in future research. First, our study only included monocytes from HDs and MM patients, which may not provide a complete understanding of monocyte‐myeloma cell interactions and monocyte‐immune cell interactions within the TME and under physiological conditions. It is important to investigate the potential contributions of MM cells and other immune cells, such as DCs, macrophages, NK cells and T cells, to the excessive IFN response observed in MM monocytes. Further investigations are needed to elucidate the specific roles of these cell types in shaping the stress signature of MM monocytes. Second, all the results in our study were derived from scRNA‐seq data, and protein‐level intervention studies were not performed. While our co‐culture experiments provide preliminary functional support, the findings remain largely correlative. More detailed mechanistic analyses and in vivo functional validation will be required to confirm the biological significance of monocyte‐associated immune suppression and to clarify the specific role of the excessive IFN response in MM pathogenesis and progression. These limitations are partly due to the limited number of monocytes available in the BM and PB of MM patients. Future studies should overcome this weakness by incorporating protein‐level analyses to investigate the functional consequences of the excessive IFN response in MM development and disease progression. Furthermore, while computational trajectory modeling offers valuable insights into potential monocyte differentiation paths, it remains an inferential approach based on transcriptional similarity rather than direct lineage evidence. Thus, the conclusions drawn from such modeling should thus be interpreted with caution. Addressing these criticisms and conducting further investigations will provide a more comprehensive understanding of the complex interactions between monocytes, MM cells, and other immune cells in the TME and shed light on the role of the excessive IFN response in the pathogenesis of MM.

In summary, our unbiased scRNA‐seq analysis successfully elucidated the identity, functional characteristics, and differentiation pathways of BM and PB monocytes in both HD and MM patients. Furthermore, we investigated the sequential changes occurring in BM monocytes after induction therapy in MM patients, highlighting the potential alleviation of chronic activation signals in the MM TME by the treatment. The comprehensive data generated in this study could serve as a valuable single‐cell resolution atlas, offering further insights into potential therapeutic interventions targeting monocytes in the context of MM. These findings paved the way for future investigations aimed at improving treatment strategies for MM patients by modulating the behavior and function of monocytes.

## 4 Experimental Section

### Human Samples

Heparin‐anticoagulated PB samples or BM aspirates were collected from seven NDMM patients and HDs. Paired BM aspirates were obtained from 10 MM patients at the time of diagnosis and after induction therapy (Tables S1 and S8, Supporting Information). All samples were collected at the Institute of Hematology & Blood Diseases Hospital, Chinese Academy of Medical Sciences & Peking Union Medical College in Tianjin, China.

PB and BM samples were processed within 2 h after collection. Peripheral blood mononuclear cells (PBMCs) and bone marrow mononuclear cells (BMMCs) were isolated using Ficoll‐Paque PLUS (Cytiva, Sweden) by standard density gradient centrifugation. Briefly, samples were mixed 1:1 with Ficoll‐Paque PLUS and centrifuged at 1800 rpm for 30 min at room temperature without brake. The mononuclear cell layer at the interface was carefully collected, washed twice with PBS, and resuspended in complete RPMI‐1640 medium. Cell viability was assessed by trypan blue exclusion using a hemocytometer, and only samples with >95% viable cells were used for flow cytometry and single‐cell RNA sequencing.

To minimize potential confounding factors, particularly those related to activation of IFN pathways, patients with documented active infections, recent antimicrobial therapy (within 4 weeks prior to sample collection), or clinical/laboratory signs of acute inflammation (e.g., fever, elevated C‐reactive protein, leukocytosis) were excluded from the study. The same exclusion criteria were applied to both MM patients and HDs.

All patients included in this study were enrolled in the National Longitudinal Cohort of Hematological Diseases in China (ClinicalTrials.gov identifier: NCT04645199). The study was approved by the local institutional ethics committees under the leadership of the Institute of Hematology & Blood Diseases Hospital, Chinese Academy of Medical Science & Peking Union Medical College (Certificate: IIT2020023‐EC‐1). All MM patients and HDs provided informed consent before enrollment, following the guidelines of the Declaration of Helsinki.

### Flow Cytometry and Cell Sorting

To obtain purified monocytes for sequencing, freshly isolated PBMCs and BMMCs were subjected to antibody staining at a temperature of 4 °C for a duration of 30 min. The antibodies used for staining were as follows: FITC‐labeled anti‐human CD14 (325604, HCD14, BioLegend), PE‐labeled anti‐human CD16 (980102, 3G8, BioLegend), APC‐labeled anti‐human CD3 (989998, OKT3, BioLegend), anti‐human CD19 (302212, HIB19, BioLegend), and anti‐human CD56 (981204, 5.1H11, BioLegend). Monocytes were then separated using fluorescence‐activated cell sorting (FACS) performed with a FACSAria III cell sorter (BD Biosciences) and BD FACSDiva version 5.0. The gating strategy can be found in Figure S1A (Supporting Information). Subsequently, cell counting, and viability assessment were conducted under microscopy using trypan blue, and only samples with viabilities exceeding 90% were utilized for library construction and sequencing. In the case of paired BM samples obtained from MM patients at the time of diagnosis and after induction therapy, monocyte purification was not performed, and the entire set of BMMCs was subjected to sequencing.

### Single‐Cell RNA Library Construction and Sequencing

The Single Cell 3' Library Kit V3 (manufactured by 10x Genomics, CA, USA) was utilized to capture individual cells for cDNA synthesis. The cells were subsequently barcoded and prepared for library construction following the instructions provided by the manufacturer. After preparation, the indexed cDNA libraries were sequenced on an Illumina NovaSeq 6000 platform using paired‐end mode. The sequencing was performed by Novogene Co., Ltd. (Beijing, China) as per the manufacturer's instructions, aiming for a sequencing depth of ≈20,000 reads per cell.

### Cell Culture

The human monocytic cell line THP‐1 (RRID: CVCL_0006), the multiple myeloma (MM) cell line NCI‐H929 (RRID: CVCL_1600), and RPMI‐8226 (RRID: CVCL_0014) were used in this study. All cell lines were obtained from the American Type Culture Collection (ATCC; THP‐1: TIB‐202, NCI‐H929: CRL‐9068, RPMI‐8226: CCL‐155). Cells were cultured in RPMI‐1640 medium supplemented with 10% fetal bovine serum (FBS) and 1% penicillin‐streptomycin at 37°C in a humidified incubator with 5% CO_2_. All cell lines were routinely tested for mycoplasma contamination using the MycoAlert Mycoplasma Detection Kit (Lonza) and confirmed negative prior to use in experiments. For MM cell–monocytes coculture experiments, monocyte cell line THP1 was cultured with MM cell lines at a 1:1 ratio. As a control, monocytes and MM cells were cultured alone in RPMI‐1640 medium. Twenty‐four hours after coculture, CD138+ MM cells and monocytes were separated using CD138+ magnetic beads (Miltenyi Biotec, Paris, France) for downstream analyses. Seventy‐two hours after coculture, conditioned media (CM) were collected from cocultured cells while CM from culture alone MM cells were used as a control. In the indicated experiments, neutralizing antibodies against IFNAR1 (2 µg mL^−1^; MA5‐23630, Thermo Fisher Scientific) or matched IgG isotype control (2 µg mL^−1^; 294815, MedChemExpress) were added 30 min prior to CM treatment. Cell viability and proliferation of MM cells were assessed using the CellTiter‐Glo Luminescent Cell Viability Assay (no. G7572; Promega), which quantifies ATP levels as an indicator of metabolically active and proliferating cells.

### Data Processing and Quality Control of scRNA‐seq Data

The initial step involved processing the raw sequencing data, which was then aligned to the GRCh38 human reference genome using the CellRanger pipeline (version 3.1.0, 10x Genomics, CA, USA). Default parameters were used for the alignment process. Following alignment, quality control procedures were performed on the datasets using Seurat (version 4.0.0).^[^ ^51^ ^]^ Low‐quality cells were identified and excluded from the analysis based on the following criteria: (i) single cells with a gene count ranging from 200 to 4,000 and containing less than 10% mitochondrial unique molecular identifiers (UMIs) were retained; (ii) cell doublets were detected using the DoubletFinder package (version 2.0.3) with default parameters,^[^ ^52^ ^]^ and (iii) genes that were expressed in at least five cells were selected for subsequent assay.

### Dimensionality Reduction, Cell Clustering, and Visualization

Following the removal of low‐quality single cells, the raw expression matrix underwent normalization using the NormalizeData function. Subsequently, the ScaleData function was applied to eliminate cell‐to‐cell variation in gene expression. Principal component analysis (PCA) was then employed for linear dimensionality reduction, utilizing the top 2000 variable genes. To determine the appropriate number of principal components for downstream clustering, the ElbowPlot function was utilized. For the integration of PB or BM datasets from both HDs and MM patients, the Harmony package (version 0.1.1) was employed with default parameters.^[^ ^53^ ^]^ The FindNeighbors function was then adopted to calculate shared nearest neighbor graphs, and the Louvain algorithm, along with uniform manifold approximation and projection (UMAP) embedding in a 2D space, was applied to cluster the single cells.

Cell types were identified by analyzing the average expression levels of well‐established markers, such as *NKG7*, *CD3D*, and *CD3E* [natural killer (NK) cells and/or T cells], *HBA1* (erythrocytes), *CD79A*, *MS4A1*, and *CD19* (B cells), *CD14* and *FCGR3A* (monocytes), *MPO* and *CD34* (hematopoietic stem and progenitor cells, HSPCs), *FCGR3B* (neutrophils), and *C1QA* (macrophages), as previously described.^[^ ^8^, ^9^, ^19^, ^54^ ^]^ Subsequently, monocytes were isolated based on the expression of known marker genes CD14 and FCGR3A, followed by a reclustering process. The same methodologies and parameters described earlier were employed to cluster monocytes into seven clusters from both BM and PB, with a resolution parameter set at 0.3 for each.

### Identification of Differentially Expressed Genes and Pathway Enrichment Analysis

To identify differentially expressed genes (DEGs), it employed the FindMarkers or FindAllMarkers function, utilizing normalized data and the following parameters: test.use = “wilcox” and logfc.threshold = 0.25. The p‐values were adjusted using the Bonferroni correction method, taking into account the total number of genes in the dataset. DEGs with adjusted p‐values below 0.05 were considered significant and selected for downstream analysis. For Gene Ontology (GO) analysis or Ranked Gene Set Enrichment Analysis (GSEA), the clusterProfiler package (version 4.2.2),^[^ ^55^ ^]^ or the fgsea package (version 1.24.0) was utilized with default parameters. These tools allowed us to perform functional enrichment analysis to identify enriched biological processes or pathways associated with the DEGs.

### Developmental Trajectory Inference

To infer the potential trajectory of monocyte differentiation, the Monocle2 package (version 2.22.0) was employed to generate pseudotime.^[^ ^37^ ^]^ The pseudotime analysis provided insight into the developmental paths of the defined monocyte subsets across various tissues and samples. To explore these paths, it utilized the orderCells function, which allowed us to examine the progression and ordering of cells along the inferred trajectory.

### Motif Enrichment and Regulatory Network

To investigate the specific gene regulatory networks associated with each BM and PB monocyte cluster, it utilized pySCENIC (version 0.12.0) in combination with the cisTarget database.^[^ ^56^ ^]^ The gene regulatory network of monocytes was constructed using default parameters. First, a coexpression network was generated to identify potential transcription factors (TFs). Subsequently, the output coexpression modules were refined using the cisTarget databases (specifically, the hg38_10kbp_up_10kbp_down_full_tx_v10_clust database). To determine the activities of the identified regulons, each cell was scored using AUCell. This scoring method allowed us to assess the regulatory activities of the identified TFs in individual cells. From the results obtained, 10 TFs based on relevant literature were selected. These selected factors were then used to evaluate the differences in gene expression regulation between monocytes from MM and HD samples.

### Functional Analysis of Signature Genes

To determine the cell cycle phase of each cell, the CellCycleScoring function was employed. This allowed us to assign a cell cycle phase to each individual cell based on its gene expression profile. Furthermore, the expression of gene signatures relevant were calculated to specific functions using the AddModuleScore function in Seurat. These gene signatures were derived from the Molecular Signatures Database, accessible at https://www.gsea‐msigdb.org/gsea/index.jsp, or manually defined based on relevant literature. The specific gene signatures used in our analysis can be found in Table S11 (Supporting Information).

### Statistical Analysis

All statistical analyses were conducted using R (version 4.1.3, R Foundation, Vienna, Austria). The identification of DEGs was performed using the two‐sided Wilcoxon rank‐sum test. In our study, all boxplots were generated, and the significance of differences between conditions was assessed using the Wilcoxon rank‐sum test (two‐sided), utilizing the ggpubr package (version 0.6.0). Unless specified otherwise, all in vitro analysis was verified using a minimum of three independent experiments. Statistical significance was determined using a threshold of *p*‐value < 0.05. The following symbols were used to indicate the level of significance: “ns” for not significant, * for *p*‐value < 0.05, ** for *p*‐value < 0.01, *** for *p*‐value < 0.001, and **** for *p*‐value < 0.0001.

### Ethics Approval Statement

The study received approval from the local institutional ethics committees of the IH & BDH, CAMS & PUMC (Certificate: IIT2020023‐EC‐1).

### Patient Consent Statement

All the patients provided informed consent in compliance with the Declaration of Helsinki.

## Conflict of Interest

The authors declare no conflict of interest.

## Author Contributions

J.C. and J.W. contributed equally to this work as co‐first authors. J.C., J.W., L.Q., and G.A. contributed to the conceptualization and design of the project. J.C. and X.M. performed flow cytometry sorting and other experiments. J.C. and X.L. designed and performed the single‐cell bioinformatic analyses. J.C. and J.W. conductedin vitro experiments and data analysis. L.W., X.M., R.L., W.Y., J.X., J.Z., C.D., S.D., and Y.X. collected clinical data and participant samples. J.C., J.W., L.Q., and G.A. wrote and revised the manuscript. S.Y., D.Z., X.G., T.C., L.Q., and G.A. supervised the study. All authors critically reviewed and approved the manuscript before submission.

## Supporting information



Supporting Information

Supporting Table

## Data Availability

The data that support the findings of this study are openly available in the Genome Sequence Archive in the National Genomics Data Center at https://ngdc.cncb.ac.cn/gsa/ under the accession numbers HRA005045 and HRA005218.
