# Supplementary material for: Type I Interferon Pathway Activation Disrupts Monocyte Maturation and Enhances Immune Evasion in Multiple Myeloma
Source: Adv Sci (Weinh). 2025 Nov 30;13(9):e10816. doi: 10.1002/advs.202510816 (PMC12904021; doi:10.1002/advs.202510816)
Supplement: Supplementary file 1 — Supporting Information [file ADVS-13-e10816-s001.docx]

**Supplementary Information for**

**Type I Interferon Pathway Activation Disrupts Monocyte Maturation and Enhances Immune Evasion in Multiple Myeloma**

*Jian Cui#, Jingwei Wang#, Xiaoyun Li, Lina Wang, Xuehan Mao, Rui Lyv, Wenqiang Yan, Jingyu Xu, Jieqiong Zhou, Chenxing Du, Shuhui Deng, Mu Hao, Yan Xu, Shuhua Yi, Dehui Zou, Tao Cheng, Xin Gao, Lugui Qiu*, Gang An**

***Corresponding Authors:**

Gang An, M.D., Ph.D.

Email: angang@ihcams.ac.cn

Lugui Qiu, M.D.

Email: qiulg@ihcams.ac.cn

**Contents:**

Figure S1-S8.


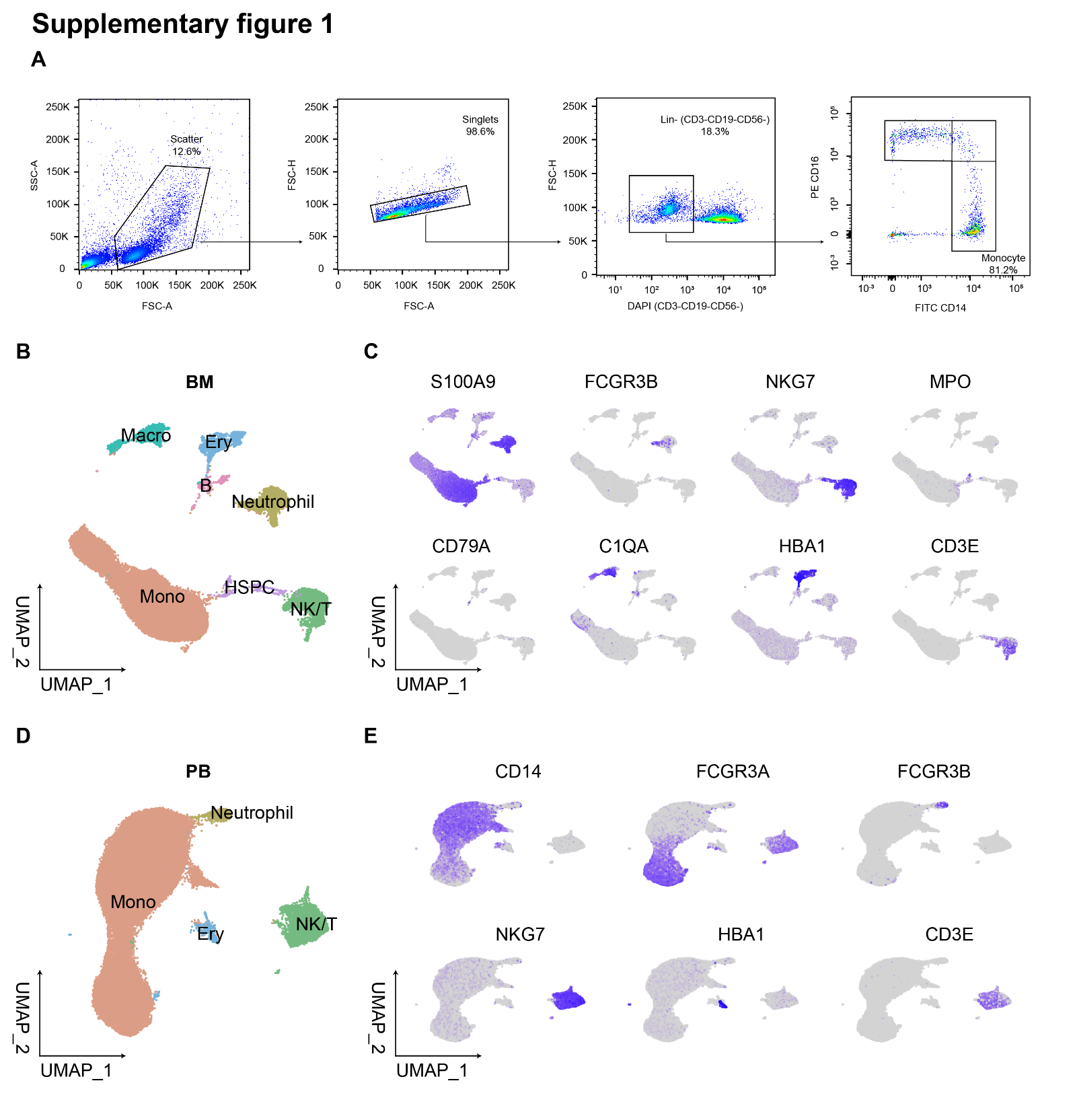


**Figure S1. scRNA-seq Analysis of Monocytes from Healthy Donors and Multiple Myeloma Patients.**

**A)** Fluorescence-activated cell sorting (FACS) strategy for human BM and PB monocytes after doublet exclusion from 1 representative patient. Plots were generated using FlowJo software. (See Experimental Section). **B)** Uniform manifold approximation and projection (UMAP) of 40,160 cells from bone marrow (BM) of healthy donors (HD) and multiple myeloma (MM) patients and colored by cell type. **C)** Expression of unique genes specifically distinguished each cluster and associated them with monocytes (Mono) (*CD14* and *FCGR3A*), neutrophils (*FCGR3B*), NK cells and T cells (NK/T) (*NKG7* and *CD3E*), hematopoietic stem progenitor cells (HSPC) (*MPO*), B cells (*CD79A*), macrophages (Macro) (*C1QA*), erythrocytes (Ery) (*HBA1*), respectively. **D)** UMAP plot of 90,575 cells from peripheral blood (PB) of HD and MM patients colored by cell type. **E)** Expression of unique genes specifically distinguished each cluster and associated them with monocytes (Mono) (*CD14* and *FCGR3A*), neutrophils (*FCGR3B*), NK cells and T cells (NK/T) (*NKG7* and *CD3E*), erythrocytes (Ery) (*HBA1*), respectively.


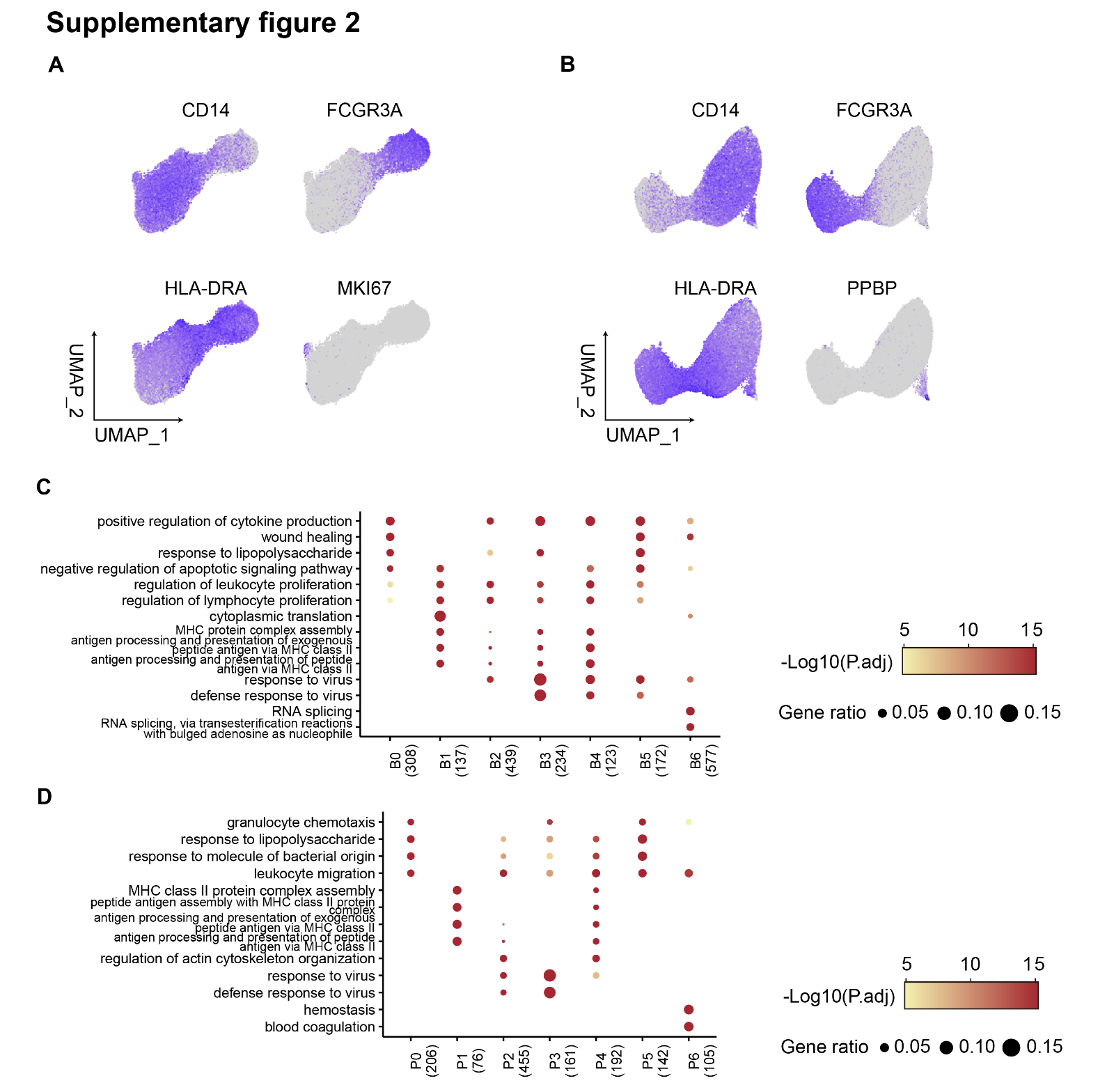


**Figure S2. Characterization of HD PB and BM Monocyte Subsets.**

**A)** Expression of unique genes specifically distinguished each BM monocyte subset, including *CD14*, *FCGR3A*, *HLA-DRA* and *MKI67*. **B)** Expression of unique genes specifically distinguished each PB monocyte subset, including *CD14*, *FCGR3A*, *HLA-DRA* and *PPBP*. **C, D)** GO analyses of cluster-based DEGs for each BM (C) or PB (D) monocyte subset. Selected GO terms with Benjamini–Hochberg-corrected P values < 0.05 (one-sided Fisher's exact test) are shown and colored by gene ratio.


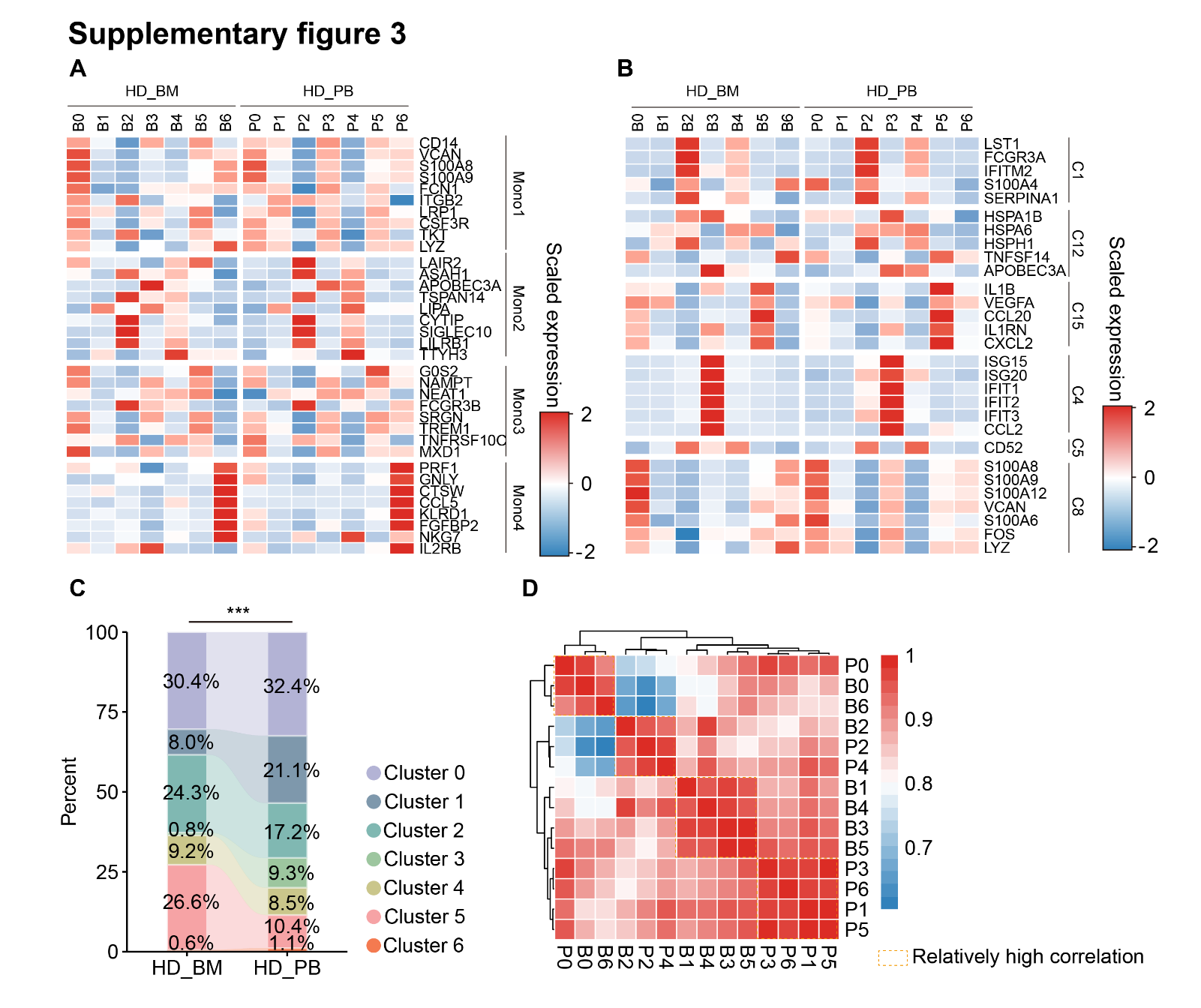


**Figure S3. Characterization of HD PB and BM Monocyte Subsets.**

**A, B)** Heatmap showing the expression of characteristic genes for monocytes, which were reported in previous articles, (A: Villani et al. *Science*. 2017;356(6335), (B: Mulder et al. *Immunity*. 2021;54(8):1883-1900.e5.). **C)** Bar plot comparing proportions of seven BM and PB monocyte subsets from HD, color coded by cell type and corresponds to Figure 1B, 1C. ****p*-value < 0.001, by two-sided χ² test. **D)** Correlogram visualizing the correlation of monocyte gene expression profiles from HD between different monocyte subsets across BM and PB tissues.


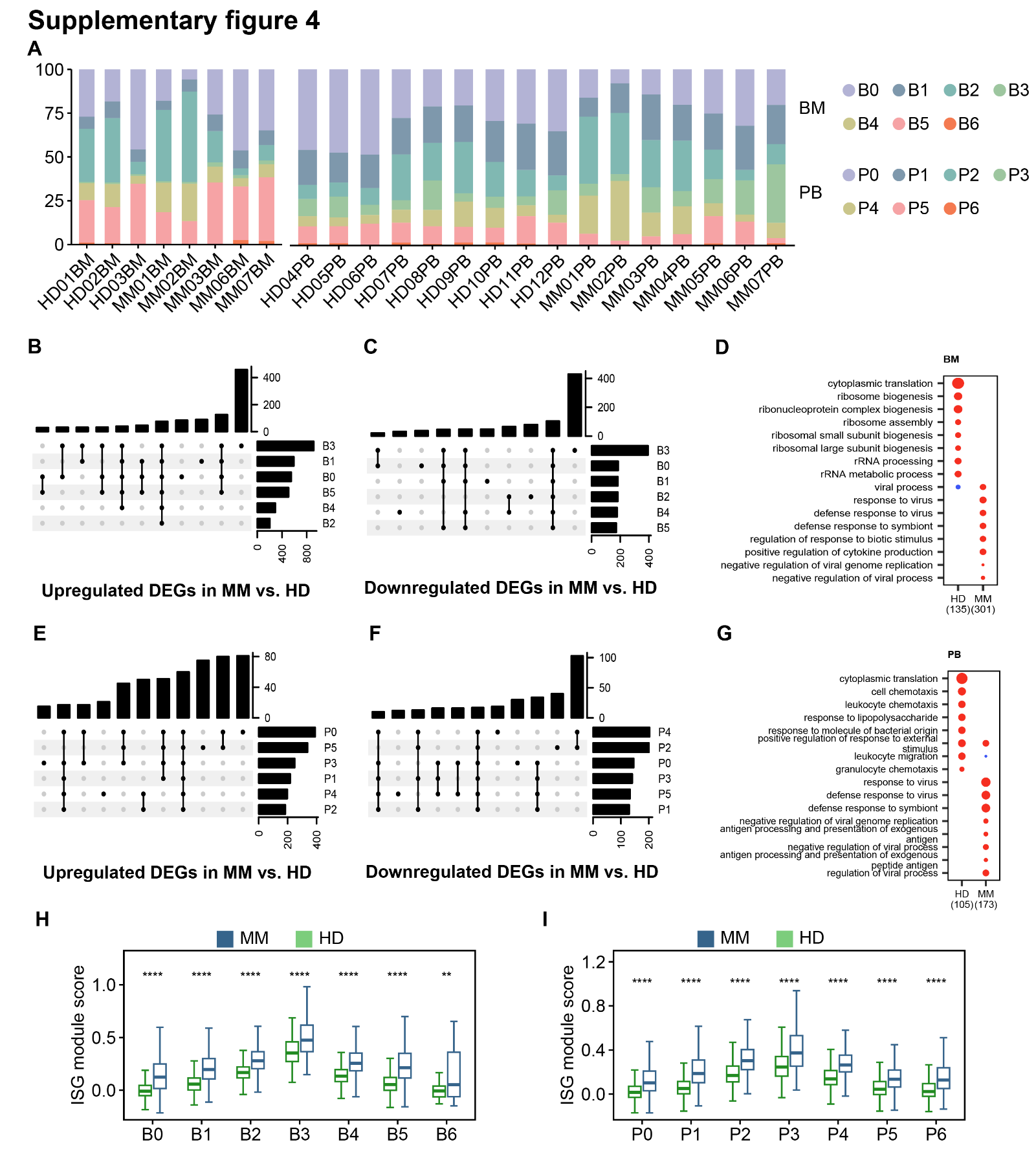


**Figure S4. Dissection of Monocyte Subpopulations of HD and MM Patients.**

**A)** The proportions of different monocyte subsets from HD and MM patients in BM and PB, respectively. **B, C)** Upset plots showing the number of upregulated (B) and downregulated (C) differentially expressed genes (DEGs) between MM and HD for each BM monocyte subset. **D)** Related to Fig. 2C, GO analyses of for DEGs of BM monocytes between MM and HD. Selected GO terms with Benjamini–Hochberg-corrected P values < 0.05 (one-sided Fisher's exact test) are shown and colored by gene ratio. **E, F)** Upset plots showing the number of upregulated (D) and downregulated (E) DEGs between MM and HD for each PB monocyte subset. **G)** Related to Fig. 2D, GO analyses of for DEGs of PB monocytes between MM and HD. Selected GO terms with Benjamini–Hochberg-corrected P values < 0.05 (one-sided Fisher's exact test) are shown and colored by gene ratio. **H, I)** Boxplots comparing the expression of IFN-stimulated gene (ISG) between HD and MM for BM (H) or PB (I) monocyte subsets. ***p*-value < 0.01, *****p*-value < 0.0001, by two-sided Wilcoxon rank-sum test.


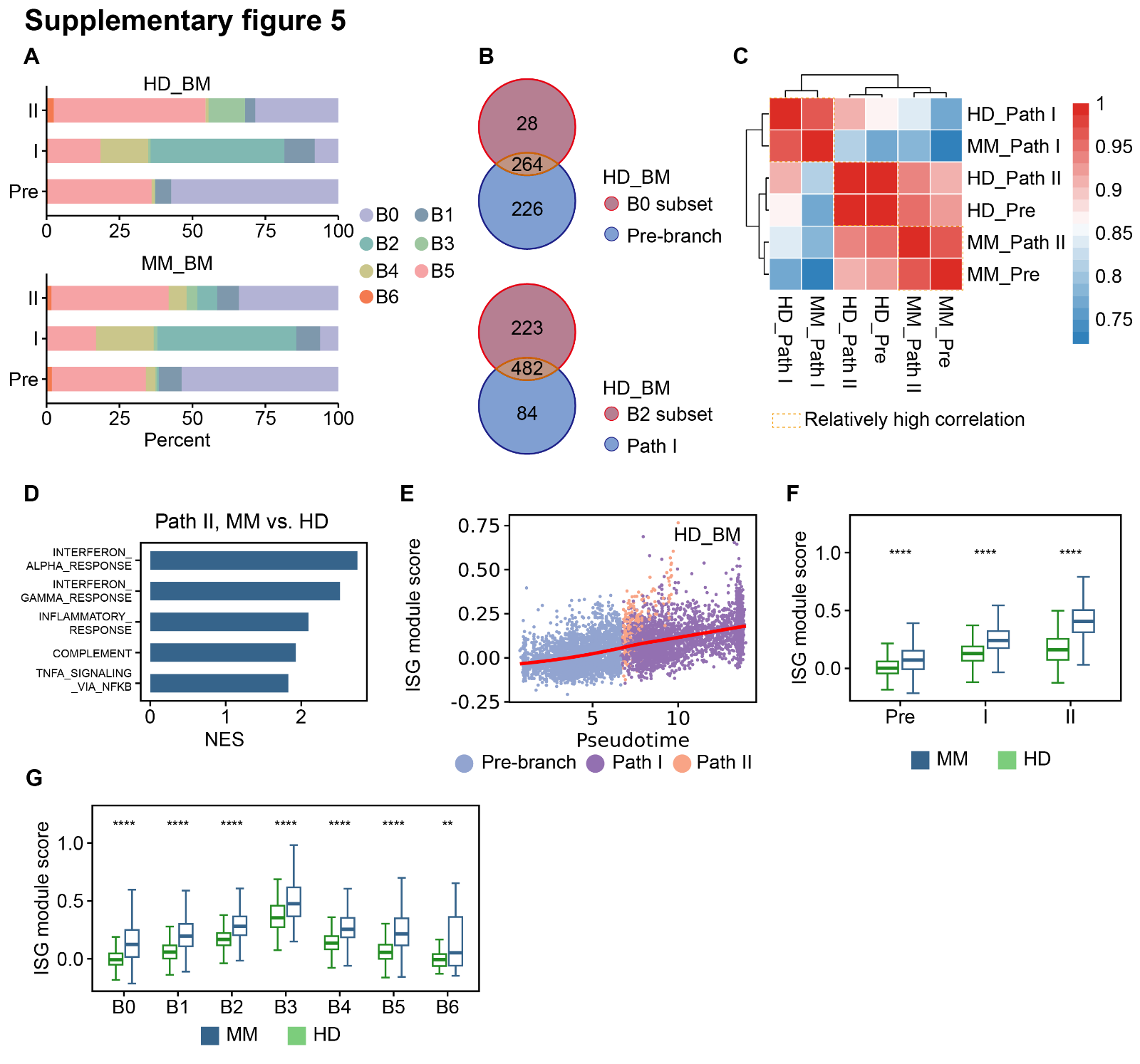


**Figure S5. scRNA-seq reveals the heterogeneity of different BM monocyte differentiation paths.**

**A)** Bar plots showing composition of different BM differentiation paths of seven monocyte subsets from HD (upper) and MM (bottom), respectively. **B)** The number of overlapped marker genes between B0 subset and Pre-branch cells (upper), or between B2 subset and Path I cells (bottom), from HD BM. **C)** Correlogram visualizing the correlation of single-cell gene expression profiles between different paths across tissues. **D)** Hallmark pathway enrichment analysis of the upregulated genes in BM Path II cells in MM compared with HD. NES, normalized enrichment score. **E)** Single-cell scatterplot showing the ISG module score of HD BM monocytes. Monocytes are ranked by the pseudotime along the x axis. Color coded by differentiation paths. **F)** Boxplot comparing the expression of ISG-related genes between HD and MM monocytes for each differentiation paths. *****p*-value < 0.0001, by two-sided Wilcoxon rank-sum test. **G)** Boxplot comparing the expression of ISG-related genes between HD and MM monocytes for each BM monocyte cluster. *****p*-value < 0.0001, ***p*-value < 0.01, by two-sided Wilcoxon rank-sum test.


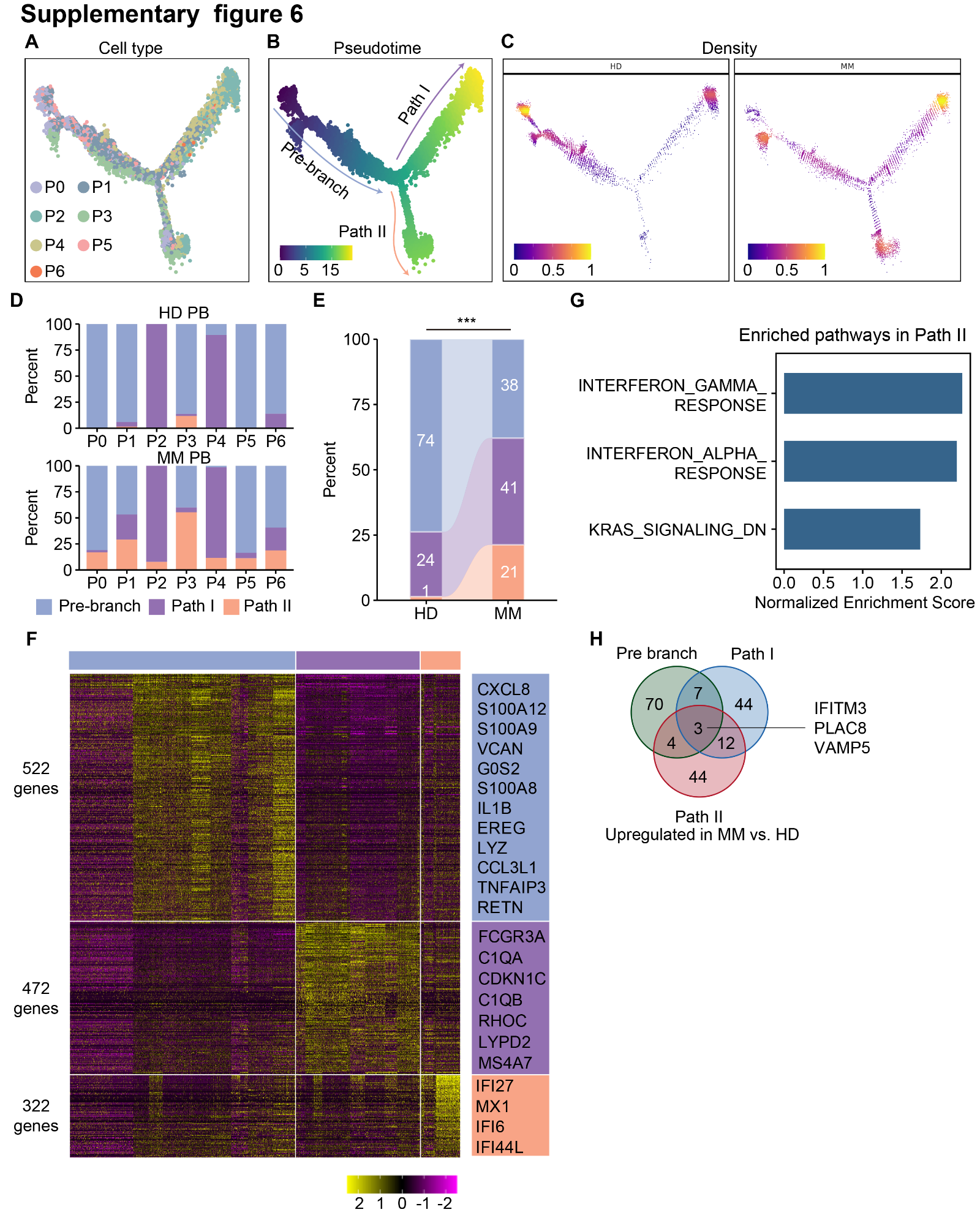


**Figure S6. PB Monocyte Differentiation Path Dysfunction in MM Patients.**

**A-C)** Trajectories predicted using the Monocle2 software for PB monocytes from HD and MM patients. Cells are color coded by monocyte subsets (A), by the pseudotime trajectory (B), and by cell density (C). **D)** Bar plots showing distribution of PB monocyte subsets in different differentiation paths from HD (upper) and MM (bottom), respectively. **E)** Bar plot comparing proportions of monocytes in three PB differentiation paths, color coded by differentiation paths and corresponds to Figure S6D. ***P < 0.001, by two-sided χ² test. **F)** Heatmap showing scaled expression of discriminative gene sets for PB monocytes for each differentiation path with average log 2-fold change ≥ 0.25 (avgLog2FC ≥ 0.25). Color bars in right margin highlight gene sets of interest. **G)** Hallmark pathway enrichment analysis of the upregulated genes in PB Path II cells compared with monocytes in other differentiation paths. **H)** The number of shared upregulated genes in MM versus HD for each PB differentiation path.


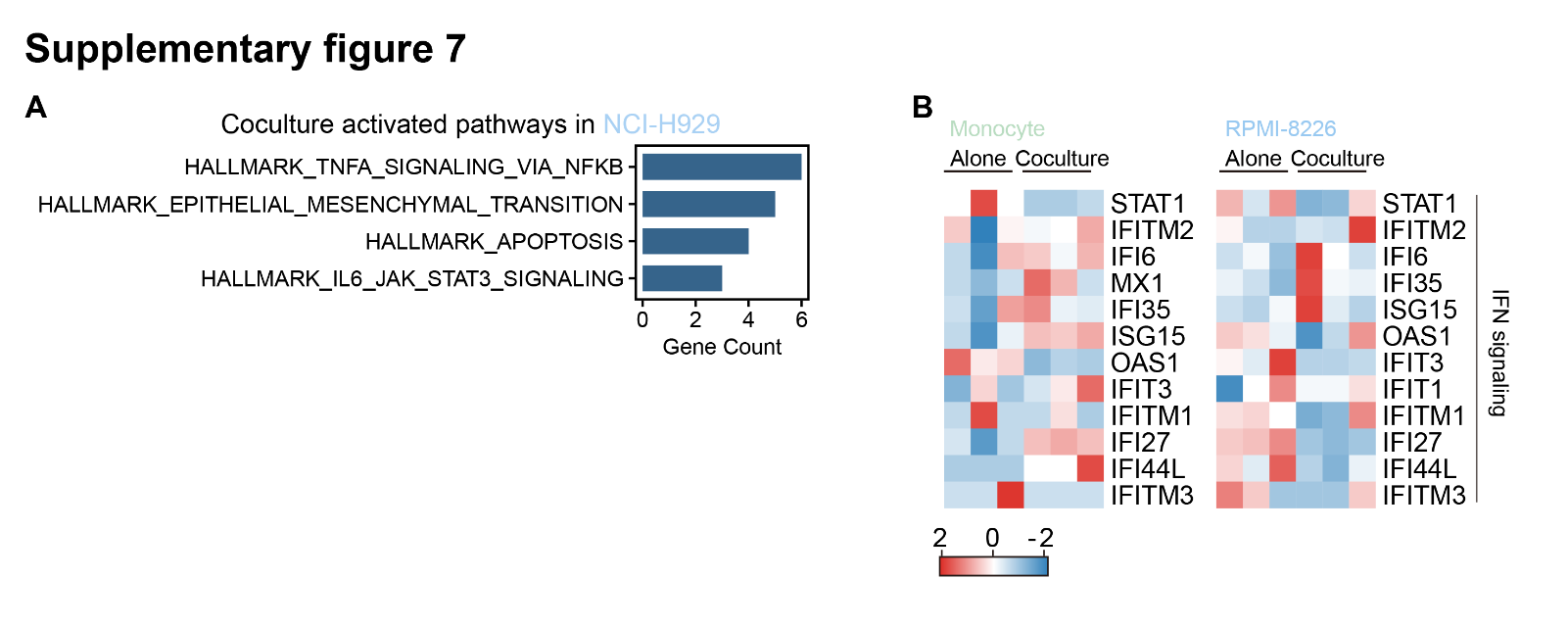


**Figure S7. In vitro analysis of IFN response activation in MM TME.**

**A**) RNA-sequencing analysis comparing monocytes-induced changes in MM cells. **B**) Heatmaps of genes in IFN signaling pathway in monocytes and MM cells.


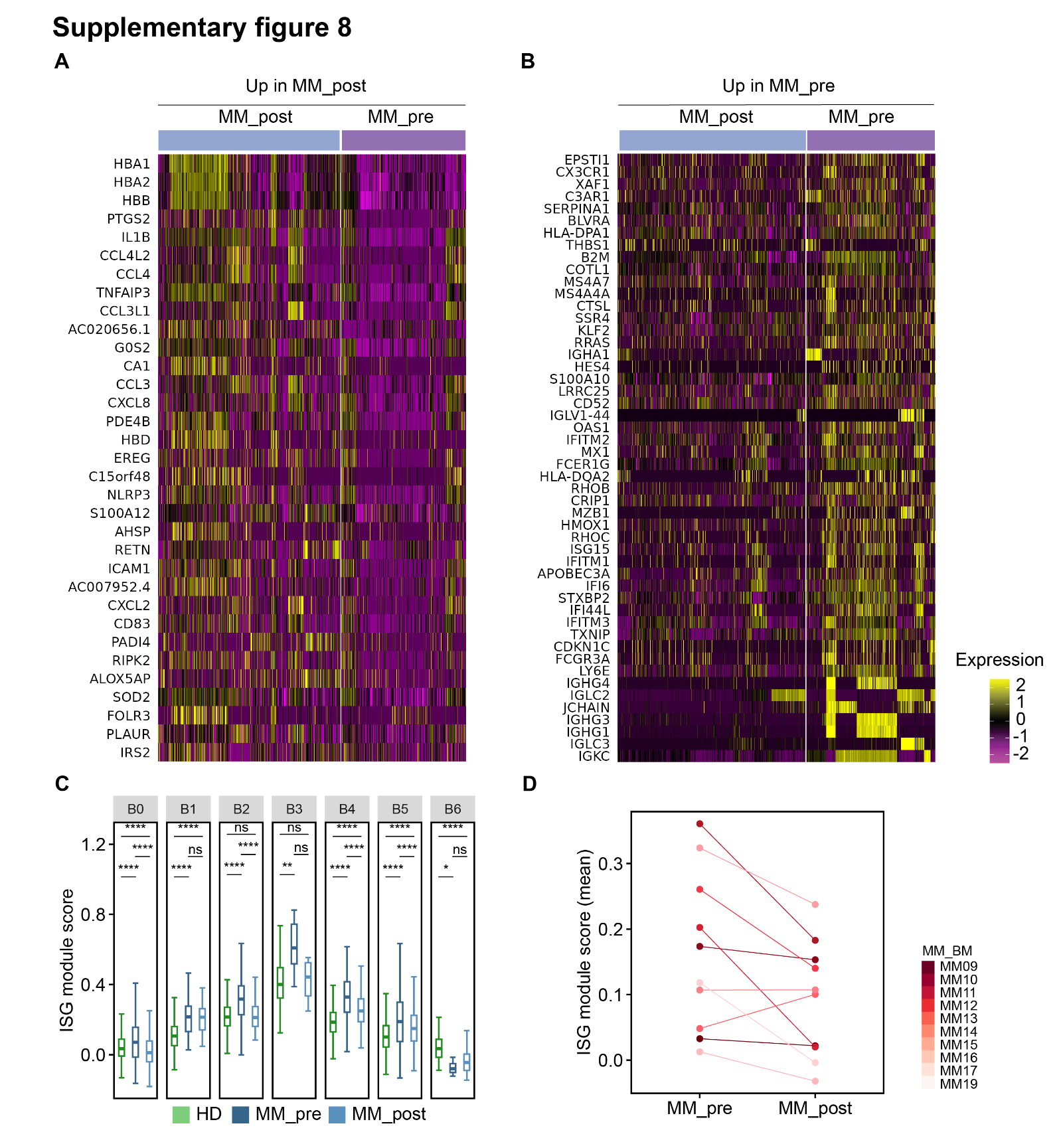


**Figure S8. Antitumor Therapy Overcomes Excessive Type I Interferon Response in BM Monocytes.**

**A, B)** Heatmaps of all DEGs that were upregulated (A) or downregulated (B) in monocytes from post-induction MM samples as compared to monocytes from MM samples at diagnosis. **C)** Boxplot comparing the expression of ISG-related genes between monocytes from HD, MM at diagnosis (MM_pre) and after induction therapy (MM_post) for each BM monocyte subset. ns, not significant, **p*-value < 0.05, ***p*-value < 0.01, *****p*-value < 0.0001, by two-sided Wilcoxon rank-sum test. **D)** Line plot comparing the mean ISG module score for each MM patient before or after induction therapy. Color coded by MM samples.
